# Expression, Delivery and Function of Insecticidal Proteins Expressed by Recombinant Baculoviruses

**DOI:** 10.3390/v7010422

**Published:** 2015-01-21

**Authors:** Jeremy A. Kroemer, Bryony C. Bonning, Robert L. Harrison

**Affiliations:** 1 Department of Entomology, Iowa State University, Ames, IA 50011, USA; E-Mails: jeremy.alan.kroemer@monsanto.com (J.A.K.); bbonning@iastate.edu (B.C.B.); 2 Current location and contact information: Monsanto Company, 700 Chesterfield Parkway West, Chesterfield, MO 63017, USA; 3 USDA-ARS Beltsville Agricultural Research Center, Invasive Insect Biocontrol & Behavior Laboratory, 10300 Baltimore Ave, Beltsville, MD 20705, USA

**Keywords:** baculovirus, genetic modification, crop protection, pest management, neurotoxins, enzymes

## Abstract

Since the development of methods for inserting and expressing genes in baculoviruses, a line of research has focused on developing recombinant baculoviruses that express insecticidal peptides and proteins. These recombinant viruses have been engineered with the goal of improving their pesticidal potential by shortening the time required for infection to kill or incapacitate insect pests and reducing the quantity of crop damage as a consequence. A wide variety of neurotoxic peptides, proteins that regulate insect physiology, degradative enzymes, and other potentially insecticidal proteins have been evaluated for their capacity to reduce the survival time of baculovirus-infected lepidopteran host larvae. Researchers have investigated the factors involved in the efficient expression and delivery of baculovirus-encoded insecticidal peptides and proteins, with much effort dedicated to identifying ideal promoters for driving transcription and signal peptides that mediate secretion of the expressed target protein. Other factors, particularly translational efficiency of transcripts derived from recombinant insecticidal genes and post-translational folding and processing of insecticidal proteins, remain relatively unexplored. The discovery of RNA interference as a gene-specific regulation mechanism offers a new approach for improvement of baculovirus biopesticidal efficacy through genetic modification.

## 1. Introduction

Baculoviruses have been formulated and deployed as biopesticides to control pest insects since the 19th century [1]. Baculovirus infection often results in the death of the host insect, and baculoviruses cause naturally occurring epizootics in populations of their hosts. As members of an insect-specific virus family, *Baculoviridae* [2,3], that contains no species causing disease in vertebrates, the baculoviruses are exceedingly safe options for pest control. Host specificity among the baculoviruses is even more stringent than that of other insect pathogens, such as entomopathogenic fungi and the bacterium *Bacillus thuringiensis*, and applications of baculoviruses tend to have little or no impact on non-target insects [4].

**Figure 1:**
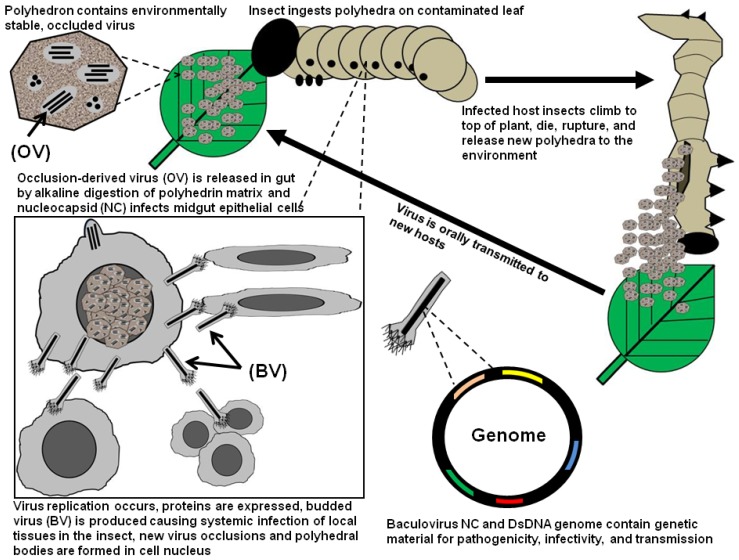
Representation of the baculovirus life cycle.

The preparation and use of baculoviruses as pest control agents have been further facilitated by the fact that baculoviruses embed infectious particles in proteinaceous occlusion bodies (OBs) that confer a level of stability to virus and can be dispersed with the same equipment and methods used for applying chemical pesticides [5,6]. These OBs, also known as polyhedra (for species of the genera *Alphabaculovirus*, *Gammabaculovirus*, and *Deltabaculovirus*) or granules (for species of the genus *Betabaculovirus*), occur in environments where larvae of the host feed. The OBs of alphabaculoviruses and betabaculoviruses have been used most frequently in biopesticide formulations to control pestiferous caterpillars of the order Lepidoptera (moth and butterflies). Larval lepidopteran hosts of alphabaculoviruses and betabaculoviruses ingest OBs while feeding on foliage (Figure 1). The paracrystalline matrix of the OBs, consisting of the viral protein polyhedrin (*Alphabaculovirus*) or granulin (*Betabaculovirus*), dissolves in the alkaline environment of the larval midgut [7]. The virions liberated from the dissolved OBs, which are referred to as occlusion-derived virus (ODV) establish primary infection of the columnar epithelial cells of the larval midgut [8,9]. For alphabaculoviruses and many betabaculoviruses, infection spreads to other host tissues by way of a second type of virion produced during viral replication known as the budded virus, or BV [10] (Figure 1). Infection and viral replication is accompanied by a characteristic disease referred to as nuclear polyhedrosis or granulosis. Infected cells lyse, and the internal anatomy is liquefied and the cuticle weakened by degradative enzymes encoded by the virus [11,12,13]. When the larval host dies from the viral disease, the cuticle ruptures and liberates progeny OBs produced during infection.

While baculovirus-infected host larvae become moribund and eventually die in a dramatic fashion, the time between the initial infection of the host and its death from viral disease is generally a matter of days or even weeks, during which time the host continues to feed and cause agricultural damage. The amount of such post-inoculation damage can be reduced by applying high rates of OBs, or by timing application of the OBs such that the earliest (and least damaging) developmental stages of the pest are infected and killed [14]. However, the slow speed of kill of baculoviruses has meant that baculoviruses are infrequently used for pest control. Baculoviruses that have been commercialized for use generally target prevalent and highly damaging pest species such as codling moth and gypsy moth, or pests in cropping systems where a relatively high degree of plant damage can be sustained without loss of economic value (for example, soybeans) [15].

A solution to the problem with slow speed of kill came with the development of methods for genetic engineering of baculoviruses to express foreign genes. The possibility of engineering baculoviruses to express insecticidal proteins that would kill infected pests rapidly or halt their feeding, or that exhibit other improvements (e.g., greater pathogenicity), was suggested almost as soon as the first papers on baculovirus-mediated foreign gene expression were published [16]. Since then, a prodigious amount of research has been carried out by many laboratories, resulting in the production of many recombinant baculoviruses that express insecticidal peptides. Larvae infected with these recombinant viruses generally die sooner than larvae infected with non-recombinant wild-type viruses, due to the toxicity of the encoded insecticidal protein rather than the pathology of the infection itself. In general, the viruses with which this work has been carried out tend to be alphabaculoviruses for which cell lines that support viral replication are available, as procedures for genetic modification of baculoviruses rely upon susceptible cell lines. In particular, strains of the baculoviruses *Autographa californica multiple nucleopolyhedrovirus* (AcMNPV) and *Bombyx mori nucleopolyhedrovirus* (BmNPV) are models of baculovirus molecular biology research and have subsequently been the starting points for many of the efforts to produce recombinant baculoviruses with improved speed of kill.

This review focuses on the generation and laboratory evaluation of recombinant baculoviruses that express insecticidal peptides, with an emphasis on the factors involved in expression of insecticidal peptides during viral infection and replication that influence the pesticidal properties of recombinant baculoviruses. Another type of genetic modification of baculoviruses that has resulted in an improvement in their insecticidal performance involves the deletion of selected genes, such as the *egt* gene [17,18]. For information on this sort of modification, as well as other facets of wild-type and recombinant baculovirus biopesticides relating to production, formulation, field testing, safety assessment, and application, the readers are referred to the comprehensive treatments of these subjects by Hunter-Fujita *et al.* [19] and Kamita *et al.* [20,21].

## 2. Insecticidal Peptides Expressed by Recombinant Baculoviruses

Insecticidal peptides that have been used to improve the pesticidal performance of baculoviruses include neurotoxic peptides that interfere with axonal membrane function, enzymes and peptide hormones that regulate the physiology of the host insect, and degradative enzymes (proteases and chitinases) that target extracellular structures, along with a selection of other peptides that do not fit into the above categories. In general, these insecticidal peptides exert their influence at the organismal level, rather than the cellular level, and thus do not interfere with baculovirus infection and replication. Different laboratories have assessed the efficacy of a given insecticidal protein in different ways. Some recombinant viruses retained an intact polyhedrin gene (*polh*) and were capable of producing OBs, which could be fed to host larvae, while other recombinant viruses lacked *polh* and were usually used in bioassays in which BV were injected into the hemocoel or “pre-occluded” ODV were fed to larvae [22]. Bioassays of recombinant viruses have featured a plethora of different host species and developmental stages, and different methods for estimating the survival time of infected larvae from bioassay data have been used [23].

### 2.1. Neurotoxins

Insect-specific peptide neurotoxins from the venoms of invertebrates were among the first insecticidal peptides to be inserted into baculoviruses. These toxins disrupt ion conductance across axonal membranes and cause paralysis. Many of the neurotoxic peptides that have been expressed using baculoviruses are small (approximately 58–70 amino acids), and it was thus relatively easy to make synthetic versions of the coding sequences for these peptides with a codon distribution that was favorable for expression in insect cells and with heterologous signal peptide sequences for efficient secretion from infected cells. Table 1 lists the recombinant baculoviruses engineered with neurotoxin genes, with the promoters and signal peptides used to mediate their expression and the degree of improvement in bioassays relative to control viruses. The viruses are listed in order of their chronological appearance in the literature, although some viruses (e.g., AcUW2(B).AaIT) have been used in multiple studies, sometimes under a different name.

**Table 1:** Recombinant baculoviruses that express peptide neurotoxins.

Recombinant Baculovirus	Insecticidal Peptide	Parental Virus/Host Species ^1^	Promoter	Signal Peptide	Improvement *vs*. Control ^2^	Reference ^3^
vBeIt-1	BeIT (scorpion)	AcMNPV/*T. ni*, *G. mellonella*	*polh*	None	No larval paralysis	[24]
vBeIt-2	BeIT	AcMNPV/*T. ni*, *G. mellonella*	*polh*	Human β-interferon	No larval paralysis	[24]
vBeIt-3	Polyhedrin-BeIT fusion	AcMNPV/*T. ni*, *G. mellonella*	*polh*	None	No larval paralysis	[24]
BmAaIT	AaIT(scorpion)	BmNPV/*B. mori*	*polh*	bombyxin	40%	[25]
AcUW2(B).AaIT [ *p10*–AaIT; AcAaIT; vAaIT; Ac.AaIT(p10); vAcAaIT.p10]	AaIT	AcMNPV/*H. armigera, H. virescens*, *S. exigua*, *T. ni*	*p10*	bombyxin	12%–50% (*n* = 23, average 29%)	[26,27,28,29,30,31,32,33,34]
AcST-1	AaIT	AcMNPV/*T. ni*	*p10*	None	No neurotoxin secretion from infected cells	[35]
AcST-3	AaIT	AcMNPV/*T. ni*	*p10*	AcMNPV GP67	24%, 25%	[35,36]
vEV-Tox34	TxP-I (mite)	AcMNPV/*T. ni*	*polh* (LSXIV)	native	Not estimated	[37]
vETL-Tox34	TxP-I	AcMNPV/*T. ni*	ETL (AcMNPV ORF *ac49*)	native	Not estimated	[38]
vCappolh-Tox34	TxP-I	AcMNPV/*T. ni*	*vp39*/*polh* fusion	native	Not estimated	[38]
vSp-tox34	TxP-I	AcMNPV/*T. ni*, *S. frugiperda*	synXIV	native	33%–59% (*n* = 7, average 44%)	[38,39,40]
vSp-tox21A	*tox21A* gene product (mite)	AcMNPV/*T. ni*	synXIV	native	34%; 49%	[39,40]
vEV-HA5f10	*Dol m* VB (hornet)	AcMNPV/*T. ni*	*polh* (LSXIV)	native	No larval paralysis or other effect	[41]
vEV-HA5f17	*Dol m* VA(hornet)	AcMNPV/*T. ni*	*polh* (LSXIV)	native	No larval paralysis or other effect	[41]
AcLα22 (vAcLqαIT)	LqhαIT (scorpion)	AcMNPV/*H. armigera*, *H. virescens*	*polh*	native	27%–41% (*n* = 3, average 34%)	[42,43]
AcNPV/RH1	AaIT	AcMNPV/*S. exigua*	*p10*	bombyxin	31%	[27]
vSp-tox34m	TxP-I	AcMNPV/*T. ni*, *S. frugiperda*	synXIV	modified native	44%; 54%	[40]
vSp-BSigtox34	TxP-I	AcMNPV/*T. ni*	synXIV	*Sarcophaga* sarcotoxin IA	26%	[40]
vSp-tox21A/tox34	TxP-I	AcMNPV/*T. ni*	synXIV	*tox21A*	36%	[40]
vSp-DCtox34	TxP-I	AcMNPV/*T. ni*	synXIV	*Drosophila* cuticle protein II	47%	[40]
vpHSP70tox34	TxP-I	AcMNPV/*T. ni*, *S. frugiperda*	*Drosophila hsp70*	native	41%; 46%	[40]
vDA26tox34	TxP-I	AcMNPV/*T. ni*, *S. frugiperda*	DA26 (AcMNPV ORF *ac16*)	native	28%; 39%	[40]
vp6.9tox34	TxP-I	AcMNPV/*T. ni*, *S. frugiperda*	*p6.9*	native	56%–59% (*n* = 4, average 58%)	[40]
vp6.9tox34m	TxP-I	AcMNPV/*T. ni*, *S. frugiperda*	*p6.9*	modified native	53%; 54%	[40]
TnNPV-AaIT (clones 1–4)	AaIT	“TnNPV”/*T. ni*	synXIV	AcMNPV GP67	20%–43%	[44]
vMAg4p+	μ-Aga-IV (spider)	AcMNPV/*T. ni*, *S. frugiperda*	synXIV	Honeybee melittin	15%–42% (*n* = 7, average 27%)	[30,45,46]
vSAt2p+	As II (sea anemone)	AcMNPV/*H. virescens, T. ni*, *S. frugiperda*	synXIV	*Sarcophaga* sarcotoxin IA	25%–38% (*n* = 6, average 33%)	[30,45]
vSSh1p+	Sh I (sea anemone)	AcMNPV/*T. ni*, *S. frugiperda*	synXIV	*Sarcophaga* sarcotoxin IA	35%–42% (*n* = 3, average 38%)	[30,45]
*ie1*–AaIT	AaIT	AcMNPV/*H. virescens*	*hr5* enhancer/ *ie-1* promoter	bombyxin	10%	[28]
vAcTalTX-1	TalTX-1 (spider)	AcMNPV/*T. ni*, *S. exigua*, *H. virescens*	*polh*	native	18%–33% (*n* = 4, average 24%	[22]
vAcDTX9.2	DTX9.2 (spider)	AcMNPV/*T. ni*, *S. exigua*, *H. virescens*	*polh*	*T. ni* AJSP-1	7%–24% (*n* = 4, average 14%)	[22]
HzEGTp6.9tox34	TxP-I (inserted in *egt* gene)	HzSNPV/*H. zea*	*p6.9*	native	35%	[47]
HzEGThsptox34	TxP-I (inserted in *egt* gene)	HzSNPV/*H. zea*	*Drosophila hsp70*	native	32%	[47]
HzEGTDA26tox34	TxP-I (inserted in *egt* gene)	HzSNPV/*H. zea*	DA26 (AcMNPV ORF *ac16*)	native	39%	[47]
vV8EE6.9tox34	TxP-I (inserted in *egt* gene)	AcMNPV-V8/*S. frugiperda*	*p6.9*	native	38%	[48]
vV8EEHSPtox34	TxP-I (inserted in *egt* gene)	AcMNPV-V8/*S. frugiperda*	*Drosophila hsp70*	native	46%	[48]
vhsMAg4p+	μ-Aga-IV	AcMNPV/*H. virescens, S. frugiperda*, *T. ni*	*Drosophila hsp70*	Honeybee melittin	20%–71% (*n* = 11, average 47%)	[30,46]
vhsSAt2p+	As II	AcMNPV/*H. viresncens, S. frugiperda*, *T. ni*	*Drosophila hsp70*	*Sarcophaga* sarcotoxin IA	50%–61% (*n* = 7, average 55%)	[30,46]
vDASAt2	As II (in *ac16*-negative mutant)	AcMNPV/*S. frugiperda*, *T. ni*	synXIV	*Sarcophaga* sarcotoxin IA	37%–40% (*n* = 4, average 39%)	[46]
vDAhsSAt2	As II (in *ac16*-negative mutant)	AcMNPV/*S. frugiperda*, *T. ni*	*Drosophila hsp70*	*Sarcophaga* sarcotoxin IA	40%–53% (*n* = 5, average 47%)	[46]
vhsSSh1p+	Sh I	AcMNPV/*H. virescens*, *S. frugiperda*, *T. ni*	*Drosophila hsp70*	*Sarcophaga* sarcotoxin IA	45%–55% (*n* = 4, average 50%)	[30,46]
vMAg4SAt2	μ-Aga-IV + As II	AcMNPV/*S. frugiperda*, *T. ni*	synXIV (both toxins)	Honeybee melittin, *Sarcophaga* sarcotoxin IA	38%–45% (*n* = 5; average 41%)	[30,46]
vhsMAg4SAt2	μ-Aga-IV + As II	AcMNPV/*S. frugiperda*, *T. ni*	*Drosophila hsp70* (both toxins)	Honeybee melittin, *Sarcophaga* sarcotoxin IA	43%–57% (*n* = 5; average 49%)	[46]
AcLIT1.p35	LqhIT1 (scorpion)	AcMNPV/*H. armigera*	*p35*	native	16%	[49]
AcLIT1.p10 (vAcLqIT1)	LqhIT1	AcMNPV/*H. armigera*, *H. virescens*	*p10*	native	24%–28% (*n* = 3, average 27%)	[43,49]
AcLIT2.pol (vAcLqIT2)	LqhIT2 (scorpion)	AcMNPV/*H. armigera*, *H. virescens*	*polh*	native	17%–32% (*n* = 3, average 24%)	[43,49]
RcHzSNPV (RcHzLqh)	LqhIT2	HzSNPV/*H. zea*, *H. virescens*	*hr5* enhancer/ *ie-1* promoter	bombyxin	31%–44% (*n* = 11, average 38%)	[32,50]
AcTOX34.4	TxP-1 ( *tox34.4*)	AcMNPV/*T. ni*	*p10*	native	Approx. 30%–50%	[51]
BmLqhIT2	LqhIT2	BmNPV/*B. mori*	*polh*	bombyxin	Not estimated	[52]
Ro6.9AaIT	AaIT	RoMNPV/*H. virescens*, *H. zea*, *O. nubilalis*	*p6.9*	bombyxin	19%–37% (*n* = 3, average 30%)	[53]
Ro6.9LqhIT2	LqhIT2	RoMNPV/*H. virescens*, *H. zea*, *O. nubilalis*	*p6.9*	bombyxin	27%–42% (*n* = 3, average 37%)	[53]
AcUW21.AaIT	AaIT	AcMNPV/*H. virescens*	*p10*	bombyxin	27%	[53]
AcMLF9.AaIT	AaIT	AcMNPV/*H. virescens*	*p6.9*	bombyxin	25%, 26%	[53,54]
AcUW21.LqhIT2	LqhIT2	AcMNPV/*H. virescens*	*p10*	bombyxin	34%	[53]
AcMLF9.LqhIT2	LqhIT2	AcMNPV/*H. virescens*	*p6.9*	bombyxin	33%, 34%	[53,54]
HaCXW2	AaIT (inserted in the *egt* gene)	HearSNPV/*H. armigera*	*polh*	bombyxin	8%; 13% (relative to *egt*-negative control virus)	[55,56]
vAcLqIT1-IT2	LqhIT1 and LqhIT2	AcMNPV/*H. armigera*, *H. virescens*	*p10* (LqhIT1), *polh* (LqhIT2)	native	24%, 41%	[43]
vAcLqαIT-IT2	LqhαIT and LqhIT2	AcMNPV/*H. armigera*, *H. virescens*	*polh* (both toxins)	native	29%, 43%	[43]
Ac.LqhIT2(hr5/ie1)	LqhIT2	AcMNPV/*H. virescens*	*hr5* enhancer/ *ie-1* promoter	native, *Drosophila* cuticle protein II, *M. sexta* adipokinetic hormone, *L. cuprina* chymotrypsin, AcMNPV GP67, BjIT, AaIT	Not estimated	[33]
Ac.LqhIT2(hr5/39K)	LqhIT2	AcMNPV/*H. virescens*	*hr5* enhancer/*39k* promoter	bombyxin	Not estimated	[33]
Ac.LqhIT2(hr5/lef3)	LqhIT2	AcMNPV/*H. virescens*	*hr5* enhancer/*lef-3* promoter	bombyxin	Not estimated	[33]
Ac.LqhIT2(hr5/ie1)	LqhIT2	AcMNPV/*H. virescens*, *S. exigua*, *T. ni*	*hr5* enhancer/ *ie-1* promoter	bombyxin	37%–56% (n = 10, average 47%)	[33]
Ac.AaIT(hr5/ie1)	AaIT	AcMNPV/*H. virescens*, *S. exigua*, *T. ni*	*hr5* enhancer/ *ie-1* promoter	bombyxin	13%–46% (*n* = 10, average 31%)	[33]
HaSNPV-AaIT	AaIT (inserted in the *egt* gene)	HearSNPV/*H. armigera*	Tandem *polh* and *p6.9* promoters	bombyxin	4%–27% (relative to *egt*-negative control virus ; *n* = 9, average 13%); 26% relative to wild-type HearSNPV	[57,58]
Px	Poneratoxin (ant)	AcMNPV/*S. frugiperda*	*polh*	none	No improvement	[59]
SPx	Poneratoxin	AcMNPV/*S. frugiperda*	*polh*	AcMNPV GP67	16%	[59]
AcIT6	BotIT6 (scorpion)	AcMNPV/*S. littoralis*	*polh*	AcMNPV EGT	33%	[60]
vAP10IT2	LqhIT2	AcMNPV/*P. xylostella, S. exigua*, *T. ni*	*p10*	bombyxin	8%–16% (*n* = 4, average 12%)	[61,62]
vAPcmIT2	LqhIT2	AcMNPV/*P. xylostella, S. exigua*, *T. ni*	Cytomegalo-virus minimal promoter	bombyxin	10%–25% (*n* = 4, average 15%)	[61,62]
vAcAaIT.39K	AaIT	AcMNPV/*H. armigera*	*39k*	bombyxin	22%	[34]
ButaIT-NPV	ButaIT (scorpion)	AcMNPV/*H. virescens*	*p10*	bombyxin	43%	[63]
P10IT2	LqhIT2	AcMNPV/*T. ni*	*p10*	bombyxin	28%	[64]
Ppag90IT2	LqhIT2	AcMNPV/*T. ni*	HzNV-1 *pag90*	bombyxin	50%	[64]
AcMNPV- *BmK* IT	*BmK* IT (scorpion)	AcMNPV/*S. exigua*	*ie-1*	native	13%	[65]
SpltNPV-Δegt-BmK	*BmK ITa*1(inserted in *egt* gene)	SpltNPV/*S. litura*	*polh*	SpltNPV F protein	15%; 28%	[66]

**^1^ Virus abbreviations:** AcMNPV, *Autographa californica* multiple nucleopolyhedrovirus; BmNPV, *Bombyx mori* nucleopolyhedrovirus; HearSNPV, *Helicoverpa armigera* single nucleopolyhedrovirus; HzSNPV, *Helicoverpa zea* single nucleopolyhedrovirus; RoMNPV, *Rachiplusia ou* multiple nucleopolyhedrovirus; SpltNPV, *Spodoptera litura* nucleopolyhedrovirus. “TnNPV” could be either AcMNPV or *Trichoplusia ni* single nucleopolyhedrovirus. **Host abbreviations:** *B. mori, Bombyx mori*; *G. mellonella*, *Galleria mellonella*; *H. armigera*, *Helicoverpa armigera*; *H. virescens*, *Heliothis virescens*; *H. zea*; *Helicoverpa zea*; *O. nubilalis*, *Ostrinia nubilalis*; *P. xylostella*, *Plutella xylostella*; *S. exigua*, *Spodoptera exigua*; *S. frugiperda*, *Spodoptera frugiperda*; *S. littoralis*, *Spodoptera littoralis*; *S. litura*, *Spodoptera litura*; *T. ni*, *Trichoplusia ni*; **^2^** The percentage by survival time is reduced relative to a control is reported for each bioassay that includes the specified recombinant virus. If results of more than two bioassays are reported, a range of percentages is reported along with the number of bioassays featuring the virus (n) and an average percentage. “No improvement” indicates that survival times achieved with the specified recombinant baculovirus were not lower than the control virus, or there was no statistically significant difference with control virus survival times. “Not estimated” indicates that survival times were not calculated in a way that allowed for a percentage reduction in survival time to be determined; **^3^** Publications are listed that report the construction of the specified recombinant virus as well as any use of the virus in a bioassay.

The first peptide neurotoxin, and first insecticidal gene, to be expressed in a recombinant baculovirus was BeIT insectotoxin-1 from the lesser Asian scorpion, *Buthus* (now *Mesobuthus*) *eupeus* [24]. Although BeIT toxin proteins (unfused or fused to a heterologous signal peptide or part of the polyhedrin gene) were expressed in cells infected with recombinant viruses, no paralysis was observed in larvae of lepidopteran hosts *Trichoplusia ni* and *Galleria mellonella* or in larvae of the blowfly (*Sarcophaga*) injected with the virus or infected-cell extracts.

Success in improvement of the pesticidal characteristics of a baculovirus was reported in subsequent experiments using the toxins AaIT from the North African scorpion, *Androctonus australis* [25,26,28,29,34,35,36,44,53,55,56,67] and TxP-I from the straw itch mite, *Pyemotes tritici* [37,38,40,47,51]. Baculoviruses expressing these toxins caused paralysis and significantly reduced survival times in infected host larvae. The AaIT toxin in particular has been a popular choice for insertion into baculoviruses, and has been used to create a variety of different recombinant strains (Table 1).

Another neurotoxin that has been frequently expressed in baculovirus vectors is LqhIT2, originally identified from the venom of *Leiurus quinqestriatus hebraeus*, the Israeli yellow scorpion [33,49,50,52,53,61,64]. LqhIT2 and AaIT are categorized as depressant and excitatory neurotoxins, respectively, on the basis of their effect on blowfly larvae injected with the toxins [68]. While AaIT and other excitatory toxins causes an immediate and long-lasting contractile paralysis in blowfly larvae, LqhIT2 and other depressant toxins causes a short-term contractile paralysis followed by a paralysis characterized by flaccidity of the fly larvae.

Numerous other venom neurotoxins have been expressed in baculoviruses. Most of these neurotoxins have been from scorpion venoms [42,43,49,60,63,65,66], but neurotoxins from the venoms of spiders [22,30,45,46], a hornet [41], an ant [59], and a sea anemone [30,45,46] have also been inserted into baculoviruses. In most cases, paralysis of infected host larvae and a reduction in survival time is achieved with expression of these neurotoxins. Two studies have reported that co-expression of two different neurotoxins encoded by a single recombinant baculovirus can sometimes exhibit a synergistic increase in the degree of reduction in host survival time that is achieved [43,46].

### 2.2. Physiological Regulators

Another class of peptides that have been inserted into baculoviruses to improve their insecticidal performance includes hormones and enzymes involved in regulating the development, behavior, and physiological state of the host insect (Table 2). The concept underlying the use of these physiological regulators is that their overexpression, or expression of modified forms of these effectors, during baculovirus infection is expected to alter the development, behavior, or homeostasis of the insect host. The larval host subsequently succumbs more rapidly to viral infection or stops feeding sooner.

**Table 2:** Recombinant baculoviruses that express regulators of host physiology.

Recombinant Baculovirus	Physiological Regulator	Parental Virus/Host Species ^1^	Promoter	Signal Peptide	Improvement *vs*. Control ^2^	Reference ^3^
BmDH5	*M. sexta* diuretic hormone	BmNPV/*B. mori*	*polh*	*Drosophila* cuticle protein II	Approximately 20%	[25,69]
AcRP23.JHE	*H. virescens* juvenile hormone esterase (JHE)	AcMNPV/*T. ni*	*polh*	native	Not determined	[70]
AcUW2(B).JHE (AcJHE)	*H. virescens* JHE	AcMNPV/*H. virescens*, T*. ni*	*p10*	native	No improvement	[26,31,71,72,73]
vJHEEGTD	*H. virescens* JHE	AcMNPV (*egt* deletion mutant)/*T. ni*	Tandem *polh* (LSXIV) and synXIV promoters	native	No improvement relative to *egt* deletion control virus	[74]
vEHEGTD	*M. sexta* eclosion hormone	AcMNPV (*egt* deletion mutant)/*S. frugiperda*	Tandem *polh* (LSXIV) and synXIV promoters	native	No improvement relative to *egt* deletion control virus	[75]
AcJHE-SG	*H. virescens* JHE-SG mutant	AcMNPV/*H. virescens*, *T. ni*	*p10*	native	16%–31% (*n* = 3, average 25%) 16%; 29%; 31%	[31,72]
AcJHE-HK	*H. virescens* JHE-HK mutant	AcMNPV/ *H. virescens*	*p10*	native	No improvement	[31,72]
AcJHE-RH	*H. virescens* JHE-RH mutant	AcMNPV/ *H. virescens*	*p10*	native	No improvement	[72]
AcJHE-DN	*H. virescens* JHE-DN mutant	AcMNPV/ *H. virescens*	*p10*	native	No improvement	[72]
vWTPTTHM	*B. mori* prothoracicotropic hormone	AcMNPV/*S. frugiperda*	Tandem *polh* (LSXIV) and synXIV promoters	*Sarcophaga* sarcotoxin IA	No improvement	[76]
vEGT-PTTHM	*B. mori* prothoracicotropic hormone	AcMNPV ( *egt* deletion mutant)/*S. frugiperda*	Tandem *polh* (LSXIV) and synXIV promoters	*Sarcophaga* sarcotoxin IA	No improvement	[76]
AcJHE.KK ( *p10*-JHE-KK; AcUW21.JHE-KK)	*H. virescens* JHE-KK mutant	AcMNPV/ *H. virescens, T. ni*	*p10*	native	0%–25% (*n* = 7, average 13%)	[28,29,31,73]
*ie1*–JHE–KK	*H. virescens* JHE-KK mutant	AcMNPV/*H. virescens*	*hr5* enhancer/ *ie-1* promoter	native	No improvement	[28]
AcBX-PBAN	*H. zea* pheromone biosynthesis activating neuropeptide	AcMNPV/*T. ni*	*polh*	bombyxin	19%; 26%	[77]
AcJHE-29	*H. virescens* JHE-29 mutant	AcMNPV/ *H. virescens , T. ni*	*p10*	native	No improvement	[73]
AcJHE-524	*H. virescens* JHE-524 mutant	AcMNPV/ *H. virescens , T. ni*	*p10*	native	No improvement	[73]
AcJHE-KSK	*H. virescens* JHE-KSK mutant	AcMNPV/*H. virescens*	*p10*	native	17%	[31]
AcJHE-KHK	*H. virescens* JHE-KHK mutant	AcMNPV/*H. virescens*	*p10*	native	No improvement	[31]
AcHezK-II S	*H. zea* helicokinin-II (one copy)	AcMNPV/*H. zea*	*polh*	Human placental alkaline phosphatase	Not estimated	[78]
AcHezK-II M	*H. zea* helicokinin-II (two copies)	AcMNPV/*H. zea*	*polh* and *p10*	Human placental alkaline phosphatase	Not estimated	[78]

**^1, 2, 3^** See footnotes for Table 1.

The first physiological regulator to be expressed in a baculovirus and shown to have an impact on insecticidal performance was a diuretic hormone from the tobacco hornworm, *Manduca sexta* [69]. In insects, diuretic hormones regulate hemolymph volume by stimulating excretion of excess water, usually in response to feeding. Overexpression of the *M. sexta* diuretic hormone with a recombinant BmNPV increased virulence in the virus and speed of kill up to 20% over wild-type BmNPV in *B. mori* larvae injected with the viruses. A significant reduction in host survival time was also achieved with baculovirus expression of the *Helicoverpa zea* diuretic hormone helicokinin II [78].

In insects, a group of terpenoids collectively referred to as juvenile hormone (JH) regulate the outcome of molting such that a larva-to-larva molt occurs, rather than a larva-to-pupa molt. Since the larval stage of pest lepidopteran species is the developmental stage that causes plant damage, reducing the titers of JH and causing a larva-pupa molt was considered to be a worthwhile goal. Towards this end, recombinant baculoviruses have been produced that over-express the enzyme juvenile hormone esterase (JHE), which degrades JH in the hemolymph [70]. While viruses expressing the native JHE from *H. virescens* exhibited no increase or a slight increase in speed of kill [26,71,74], mutant forms of JHE showed enhancements in feeding suppression, lethality and speed of kill when inserted and expressed in a baculovirus [29,31,72,73].

Other physiological regulators that have been inserted and tested in recombinant baculoviruses include eclosion hormone, which triggers ecdysis, the emergence of a new developmental stage from the cuticle of the previous developmental stage [75]; prothoracicotropic hormone, which triggers the secretion of ecdysteroids that initiate molting [76]; and pheromone biosynthesis activating neuropeptide (PBAN), which stimulates mating pheromone biosynthesis [77]. Of these hormones, only PBAN reduced the survival time of infected larvae when overexpressed in a baculovirus.

**Table 3:** Recombinant baculoviruses that express degradative enzymes.

Recombinant Baculovirus	Degradative Enzyme	Parental Virus/Host Species ^1^	Promoter	Signal Peptide	Improvement *vs*. Control ^2^	Reference ^3^
vAcMNPV.chi	*M. sexta* chitinase	AcMNPV/*S. frugiperda*	*polh*	native	22%; 23%	[80]
AcIE1TV3.STR1	Rat stromelysin-1	AcMNPV/*H. virescens*	*hr5* enhancer/ *ie-1* promoter	native	No improvement	[54]
AcMLF9.STR1	Rat stromelysin-1	AcMNPV/*H. virescens*	*p6.9*	native	No improvement	[54]
AcIE1TV3.GEL	Human gelatinase A	AcMNPV/*H. virescens*	*hr5* enhancer/ *ie-1* promoter	native	No improvement	[54]
AcMLF9.GEL	Human gelatinase A	AcMNPV/*H. virescens*	*p6.9*	native	12%	[54]
AcIE1TV3.ScathL	*S. peregrina* cathepsin L	AcMNPV/*H. virescens*	*hr5* enhancer/ *ie-1* promoter	native	No improvement	[54]
AcMLF9.ScathL	*S. peregrina* cathepsin L	AcMNPV/*H. virescens*	*p6.9*	native	49%; 51%	[54,81]
AcMNPV-enMP2	MacoNPV-A enhancin	AcMNPV/*T. ni*	Native	None	No improvement at same effective dose as control	[82]
BacVEFPol	TnGV enhancin	AcMNPV/*T. ni*	*p10*	None	17%	[83]
AcMLF9.ScathL.CAT	*S. peregrina* cathepsin L	AcMNPV/*H. virescens*	*p6.9*	native	54%	[81]
AcMLF9.ScathL.hsp70/lacZ	*S. peregrina* cathepsin L	AcMNPV/*H. virescens*	*p6.9*	native	51%	[81]
AcBAC–polhvfgf	AcMNPV viral fibroblast growth factor (stimulates protease synthesis/activation)	AcMNPV/*S. frugiperda*, *T. ni*	*polh*	none	20%; 23%	[84]
AcMNPV-GFP-HCB-Polh	*H. armigera* cathepsin B	AcMNPV/*H. armigera*	*polh*	native	11%	[85]
HearNPV-cathL	*S. peregrina* cathepsin L (inserted in *egt* gene)	HearSNPV/*H. armigera*	Tandem *polh* and *p6.9*	native	26%; 26%	[58]
vSynScathL	*S. peregrina* cathepsin L	AcMNPV/*S. frugiperda*	synXIV	native	26%; 65%	[86]
vSynKerat	*Aspergillus fumigates* keratinase	AcMNPV/*S. frugiperda*	synXIV	native	33%; 48%	[86]

**^1, 2, 3^**See footnotes for Table 1.

### 2.3. Degradative Enzymes

Insect anatomy presents barriers to the dissemination of baculovirus infection through the body of a host insect, and enzymes that degrade these barriers have been evaluated for their capacity to improve the insecticidal performance of recombinant baculoviruses (Table 3). The first of these barriers is the peritrophic matrix, a network of chitin, glycoprotein, and proteoglycan that lines the lumen of the insect midgut. The peritrophic matrix protects the midgut epithelial cell layer from physical and chemical threats to its integrity and impedes access of pathogens (including baculoviruses) to the midgut epithelial cell layer [79].

A recombinant isolate of AcMNPV was engineered to over-express the gene for *Manduca sexta* chitinase, an enzyme that digests chitin [80]. This virus killed *S. frugiperda* larvae faster than wild-type AcMNPV in bioassays. However, it was not determined if the overexpressed chitinase had an impact on the integrity of the peritrophic matrix. Baculoviruses also encode and express their own chitinase, but this chitinase appears to target the chitin fibrils of the host cuticle, weakening the cuticle so that it will rupture more easily and release progeny occlusion bodies upon death of the host larva [13].

Granuloviruses and some group II alphabaculoviruses encode metalloproteases called enhancins that degrade mucin-like proteins in the peritrophic matrix of their hosts and increase access of ODV to the underlying epithelital cells [87,88,89]. Group I alphabaculoviruses do not contain enhancin genes. Heterologous expression of enhancin genes from the *Trichoplusia ni* GV and the *Mamestra configurata* NPV in the group I alphabaculovirus AcMNPV resulted in reductions in the doses required to kill larvae (LC_50_) [82,83]. A significant reduction in survival time was reported by del Rincón-Castro and Ibarra [83] but not by Li and coworkers [82]. The survival time reduction in the former study may have reflected the impact of a higher effective dose of the recombinant virus on speed of kill.

Beyond the midgut, the tissues of the internal anatomy are lined with extracellular sheets of proteins known as basement membranes [90]. A variety of studies have indicated that virions (specifically, the BV) of baculoviruses cannot freely diffuse through the basement membranes surrounding the midgut and other tissues [91,92,93]. Nevertheless, baculoviruses appear to have some capacity to directly penetrate the basement membrane surrounding the midgut epithelium by some unknown mechanism [9]. Baculoviruses also appear to utilize the host tracheal system as a means to escape the midgut sheath and establish infection of other tissues [94]. Specialized extensions of tracheal epidermal cells called tracheoblasts are in close proximity to midgut epithelial cells and appear to be the first targets of secondary infection by baculoviruses. Most baculoviruses encode homologues of fibroblast growth factor (FGF), a paracrine factor involved in a wide variety of host cellular and developmental processes, including cell motility and tracheal branch formation [95]. The viral FGF (VFGF), when expressed during infection, appears to mimic the host FGF in that it triggers the production and activation of proteases that digest basement membrane proteins surrounding the midgut epithelium-associated tracheoblasts [96]. While an *vfgf* mutant baculovirus killed host larvae more slowly [97], a recombinant baculovirus engineered to overexpress *vfgf* killed larvae faster than the wild-type control [84].

These results suggested that baculovirus systemic infection, along with the death of the host, can be accelerated by expressing enzymes that targeted the basement membranes posing a physical barrier to the spread of infection throughout the larval host. In an earlier study, recombinant clones of AcMNPV were produced that expressed proteases involved in the normal turnover of basement membranes [54]. Three such proteases—stromelysin-1 from rat, human gelatinase A, and a cathepsin L from the fly *Sarcophaga peregrina* (ScathL)—were evaluated for their capacity to increase speed of kill. A virus expressing ScathL exhibited the greatest reduction in host survival time. Although the ScathL protease did not appear to actually accelerate systemic infection, it did cause severe damage to basement membranes overlying the midgut, fat body and muscle tissues in infected larvae, followed by melanotic encapsulation of tissues that were no longer covered by basement membrane [93,98]. ScathL was also found to be effective in improving the insecticidal performance of different baculoviruses and against different host species [58,86]. Other proteases have also been used successfully to shorten survival time when expressed in a baculovirus [85,86].

### 2.4. Other Insecticidal Proteins

Other proteins that don’t fit into the above categories have been evaluated for the ability to shorten host survival time when expressed in baculoviruses (Table 4). Several studies have been published on the expression of the crystalline (Cry) toxins of *B. thuringiensis*. Baculovirus expression of Cry toxin genes does result in the production of toxin proteins that will kill lepidopteran larvae when infected-cell extracts are fed to them [99,100]. Since intrahemocoelic injection of Cry toxins has also been reported to cause mortality among lepidopteran larvae [101], it was thought that secretion of active fragments of the Cry toxins from baculovirus-infected cells and tissues would accelerate the onset of mortality arising from baculovirus infection. However, recombinant baculoviruses carrying full-length and truncated versions of Cry protein genes did not exhibit improvements in speed of kill when injected into larvae, even though the all versions of the toxins were insecticidal when fed to larvae [102]. In a subsequent study, the signal peptide sequence of *H. virescens* JHE was added to the N-termini of full-length and “mature” (active) Cry toxin sequences to promote secretion of the Cry toxins [27]. The “mature” Cry toxin sequence, which represented the proteolytically activated form of the toxin, proved to be cytotoxic to the extent that recombinant viruses expressing this form of the toxin could not produce OBs. The full-length toxin with the JHE signal peptide was not secreted from infected tissue culture cells, and viruses expressing this form of the toxin did not exhibit a speed of kill that was significantly different from wild-type virus. Bioassays were complicated by the presence of contaminating Cry toxin in recombinant virus OB preparations, and the researchers found it necessary to purify OBs by density gradient centrifugation. A more recent study reported a reduction in LD_50_ with a baculovirus expressing a truncated form of Cry1Ab toxin, but bioassays had been carried out with cell extracts, suggesting that the investigators in this report were measuring the oral activity of the expressed toxin rather than an improvement on the performance of the virus [103].

**Table 4:** Recombinant baculoviruses that express other insecticidal proteins.

Recombinant Baculovirus	Insecticidal Protein	Parental Virus/Host Species ^1^	Promoter	Signal Peptide	Improvement *vs*. Control ^2^	Reference ^3^
Ac(PH+)Bt	*B. thuringiensis* CryIA(c)	AcMNPV/*T. ni*	*polh*	none	Not determined	[99]
AcBtm	*B. thuringiensis* CryIA(b) (full-length)	AcMNPV/*H. virescens*	*polh*	none	No improvement	[102]
AcBt5	*B. thuringiensis* CryIA(b) (N-terminal truncated)	AcMNPV/*H. virescens*	*polh*	none	No improvement	[102]
AcBt3	*B. thuringiensis* CryIA(b) (C-terminal truncated)	AcMNPV/*H. virescens*	*polh*	none	No improvement	[102]
AcBt5/3	*B. thuringiensis* CryIA(b) (N- and C-terminal truncated)	AcMNPV/*H. virescens*	*polh*	none	No improvement	[102]
BV13T	*Z. mays* URF13	AcMNPV/*T. ni*	*polh*	native	Not estimated	[106]
Bv13.3940	*Z. mays* URF13 mutant	AcMNPV/*T. ni*	*polh*	native	Not estimated	[106]
AcNPV/JM2	*B. thuringiensis* CryIA(b) (C-terminal truncated)	AcMNPV/*S. exigua*	*p10*	none	No improvement	[27]
AcNPV/FW3	*B. thuringiensis* CryIA(b) (C-terminal truncated)	AcMNPV/*S. exigua*	*p10*	*H. virescens* juvenile hormone esterase	No improvement	[27]
vHSA50L	PBCV-1 cv-PDG (pyrimidine dimer-specific glycosylase)	AcMNPV/*S. frugiperda*, *T. ni*	*Drosophila hsp70*	none	No improvement *vs. T. ni;* 48% *vs. S. frugiperda*	[107]
vHSA50LORF	PBCV-1 cv-PDG	AcMNPV/*S. frugiperda*, *T. ni*	*Drosophila hsp70*	none	No improvement *vs. T. ni;* 41% *vs. S. frugiperda*	[107]
ColorBtrus	AcMNPV polyhedrin-*B. thuringiensis* Cry1A(c)-green fluorescent protein fusion	AcMNPV/*P. xylostella*	*polh*	None	63%	[105]

**^1, 2, 3^** See footnotes for Table 1.

A recently-pursued strategy to exploit Cry toxin expression for improvement of baculovirus insecticidal efficacy involved fusing a Cry toxin sequence to the polyhedrin gene and producing recombinant clones of AcMNPV with a native *polh* and a *polh*-*cry* chimeric sequence. Je *et al.* [104] found that a baculovirus expressing both a native polyhedrin and a polyhedrin with the green fluorescent protein fused to the C-terminus would produce fluorescent OBs in tissue culture cells, while a virus encoding only a polyhedrin-GFP fusion did not produce OBs. An immunogold EM staining experiment intended to determine if the polyhedrin-GFP fusion was incorporated in the OBs did not take background staining of the protein-rich OB matrix into account, and it is unclear from this study if the polh-GFP fusion was present in the paracrystalline matrix of the OBs, or merely associated with the OB surface or packaged within the ODV. In a subsequent study, the same authors produced a recombinant virus encoding a native polyhedrin protein and a fusion protein consisting of a Cry toxin sequence fused at the N-terminus with *polh* and at the C-terminus with GFP [105]. Bioassays with OBs produced by this recombinant generated concentration-response and time-response curves that were very similar to curves produced with equivalent quantities of purified Cry toxin. No effort was made to determine and quantify the causes of larval death in their bioassays. Given the propensity for baculovirus-expressed Cry protein to contaminate OB preparations and influence the results of bioassays [27], the possibility remains that most of the mortality in the bioassays in this study was due to toxicity from Cry peptides derived from the *polh*-*cry*-GFP fusion sequence that may have been contaminants of the OB preparation.

Other proteins that were found to reduce survival time when expressed in baculoviruses include a maize mitochondrial membrane protein [106] and a viral pyrimidine-dimer DNA repair enzyme [107].

## 3. Factors Involved in Successful Improvement of Baculovirus Pesticidal Performance with Insectidal Protein Expression

### 3.1. Choice of Promoter

Baculovirus genes can be classified on the basis of when they are transcriptionally active during the baculovirus replication cycle. Early genes are active prior to the onset of baculovirus DNA replication, and can be further sub-divided into immediate-early genes, which do not require prior protein expression to be transcribed, and delayed-early genes, which do require protein expression [108]. Late genes are transcribed concomitantly or after the beginning of viral DNA replication, and are characterized by the presence of a tetranucleotide motif, TAAG, in which transcription initiates [109]. The late genes include a sub-category, very late genes, whose transcription begins some hours after the onset of other late genes and results in the accumulation of very high steady-state levels of RNA [109].

The polyhedrin gene is a very late gene, and its strong promoter was a significant selling point for the use of baculovirus expression vectors to drive recombinant protein expression. During infection, polyhedrin accumulates to a significant proportion of the total intracellular protein in infected cells, and this high quantity is attributable to the strength of the *polh* promoter [110,111]. Thus, recombinant baculoviruses engineered with coding sequences for insecticidal proteins often used the *polh* promoter to drive expression at first [24,37,69].

However, the *polh* promoter is one of the last promoters to be activated during baculovirus infection. This can be a problem for proteins intended to act as insecticidal peptides, as these proteins need to be secreted. The secretory pathway in baculovirus-infected cells tends to deteriorate as infection proceeds, and the efficiency of protein secretion at the time that the *polh* promoter is activated is significantly lower than it is in uninfected cells [112]. Thus, it was suggested that survival time can be shortened further by using a promoter that is activated earlier than *polh* [28].

Several other viral promoters as well as cellular promoters have been investigated as possible alternatives to the *polh* promoter. The baculovirus p10 gene is also a very late gene that is expressed at high levels, but its promoter is activated a few hours earlier post-infection than that of *polh* [113]. The promoter of the late gene *p6.9*, activated after viral DNA replication but before *p10*, has also been evaluated, as research with this promoter suggests that it can drive expression of both intracellular and secreted marker proteins at higher levels than *p10* or *polh* [114]. Synthetic promoters consisting of both late and very late promoter elements were developed to take advantage of both the beneficial earlier timing of late gene transcription initiation and the quantity of transcription associated with the *polh* promoter [38,115], as have tandem arrays of late and very late promoters [57,58,74,75,76]. A plethora of baculovirus early promoters and promoters from other sources have been tested, including promoters of the baculovirus early genes *ie-1* [28,50,54,65], AcMNPV ORF *ac16* (also called DA26) [40,47], AcMNPV ORF *ac49* (also called ETL) [38], *p35* [49], *lef-3* [33], and *39k* [33,34]; and promoters from the *Drosophila melanogaster hsp70* gene [40], a minimal promoter from human cytomegalovirus (CMVm) [61], and the nudivirus HzNV-1 *pag90* promoter [64]. In some cases, the viral promoters tested were combined with the *homologous repeat* region *hr5*, which acts as an enhancer of baculovirus transcription [28,33,50,54].

A number of studies have compared the extent to which different promoters drive expression of insecticidal proteins and reduce survival time of infected hosts. In general, when levels of baculovirus-expressed neurotoxins were measured, the late and very late promoters appeared to produce noticeably more neurotoxin as assessed by western blot than the early promoters [28,40]. Likewise, the late *p6.9* promoter drove higher levels of protease expression compared to an *hr5-ie1* enhancer/promoter assembly, as measured by gel zymography and enzyme assays [54]. In some cases, higher levels of insecticidal protein expression led to shorter survival times in bioassays with viruses using the stronger late promoters compared to viruses using the weaker early or cellular promoters [28,49,54]. However, some recombinant viruses that used an early promoter for expression killed larvae as fast as, or faster than, recombinant viruses using a very late promoter (*polh* or *p10*) [33,34,40]. The relative performance of the same *hr5/ie-1* promoter construct has varied from study to study. Jarvis and coworkers [28] found that it failed to mediate a significantly faster speed of kill than the *p10* promoter even though AaIT was secreted into the medium of infected cells far earlier after infection when AaIT expression was under *ie-1* promoter control. Conversely, a similar study by van Beek and coworkers [33] found that viruses using this construct did kill some host species and developmental stages faster than viruses using the *p10* promoter. Similarly, the late *p6.9* promoter was found to mediate faster speeds of kill than the synXIV and *p10* promoters when used to drive toxin expression in recombinant viruses [40,53].

### 3.2. Choice of Signal Peptide

Since many insecticidal proteins are usually secreted, they bear a signal peptide at the N-terminus that directs the protein into the endoplasmic reticulum, which is the first step in the pathway leading to secretion from the cell. For some of the neurotoxins selected for expression in baculoviruses, the only sequence data available was of the amino acid sequence of the mature peptide (e.g., [59,63]). Such a sequence would be missing the signal peptide, which is cleaved off upon translocation of the nascent toxin peptide chain into the endoplasmic reticulum. Hence, it was necessary in such cases to add a signal peptide sequence when synthesizing the coding sequence for the neurotoxin. In addition, native signal peptides of some insecticidal proteins (Hez-PBAN, *B. mori* PTTH) did not appear to mediate processing and secretion of the encoded protein when expressed with a baculovirus, necessitating the replacement of the native sequence with a heterologous signal peptide [76,77]. In addition, an early study found that modifying the signal peptide could drive a higher level of secreted recombinant protein expression from a baculovirus vector [116].

Heterologous signal peptides from multiple sources have been used in baculoviruses expressing insecticidal proteins, including signal peptides from human beta-interferon [24], human placental alkaline phosphatase [78], *D. melanogaster* cuticle protein II [40], *B. mori* bombyxin [25], *H. virescens* juvenile hormone esterase [27], *T. ni* acidic juvenile hormone-suppressible hemolymph protein (AJSP-1; [22]), flesh fly sarcotoxin IA [40,69], honeybee melittin [45], *M. sexta* adipokinetic hormone [33], *Lucilia cuprina* (Australian sheep blowfly) chymotrypsin [33], AcMNPV GP67 [33,35,59], AcMNPV EGT [60], *Spodoptera litura* NPV EFP (envelope fusion protein) [66], and the venom neurotoxin signal peptides from the mite toxin encoded by tox21A [40] and neurotoxins AaIT, LqhIT2, and BjIT (from the scorpion *Hottentota judaicus*) [33]. In two studies, signal peptides from different sources were compared to identify a sequence that drove optimal levels of protein secretion. In a study utilizing TXP-I, the highest levels of intracellular and secreted toxin as assessed by western blot were obtained in tissue culture with the native TXP-1 signal peptide and the signal peptides of *D. melanogaster* cuticle protein II and the *tox21A* gene [40]. The recombinant viruses using these signal peptides exhibited the swiftest time to paralysis in bioassays, but the other viruses, which generated little to no detectable toxin in tissue culture, still caused paralysis in infected larvae. In a later study utilizing LqhIT2, no data on toxin protein levels were reported, but of eight signal peptides compared, five (bombyxin, adipokinetic hormone, chymotrypsin, GP67, and the native LqhIT2 sequence) mediated sufficient LqhIT2 expression to cause larval paralysis [33]. Of these five, the recombinant virus using the bombyxin signal peptide resulted in the fastest time to paralysis. Three signal peptides did not appear to drive enough LqhIT2 secretion to cause paralysis. Surprisingly, the *D. melanogaster* cuticle protein II signal peptide, which was the most effective signal peptide used with TXP-I, evidently was not effective in mediating sufficient quantities of toxin protein secretion when combined with LqhIT2 [33].

### 3.3. Translational Efficiency and Post-Translational Processing

In transgenic plants expressing *B. thuringiensis* Cry toxins, modification of the *cry* coding sequences was found to boost the quantity of Cry toxin produced [117]. A similar strategy has been followed for insecticidal proteins expressed in baculoviruses that consisted of relatively small peptides (e.g., [52,59,63,69]. Synthetic versions of the coding sequences for these insecticidal proteins were produced with a codon distribution that reflected the codon bias of the baculoviruses into which the genes were to be inserted. However, no data has been reported on whether this approach results in higher levels of insecticidal protein expression or better performance in bioassays.

As mentioned above, protein secretion in baculovirus-infected cells becomes less efficient as infection proceeds. This is likely due to a global shutdown in host gene transcription that occurs in baculovirus-infected cells [118,119]. This shutdown could also cause other problems with post-translational processing required for the functionality of insecticidal protein expressed during baculovirus infection. A study of AaIT secretion from infected cell culture revealed that, in cells infected with a baculovirus that utilized the *p10* promoter to drive expression, much of the AaIT protein was misfolded, insoluble, and inactive [120]. Co-infection with a baculovirus that expressed a chaperone that aids protein folding resulted in a larger proportion of soluble AaIT. Although there was no increase in the proportion of active AaIT in this study, the results suggest that incorporation of genes that facilitate folding and other forms of necessary post-translational processing may improve the expression of functional insecticidal protein during viral infection. Reductions in the quality and extent of protein N-glycosylation of proteins expressed late during baculovirus infection have been documented [121]. Protein N-glycosylation plays a role in protein folding and can affect the behavior and activity of a protein. Consequently, impairment of the pathway that glycosylates proteins and processes the added glycans can conceivably reduce the effectiveness of a baculovirus-expressed insecticidal protein.

### 3.4. Interaction with Host Physiology

In some cases, insecticidal proteins expressed during baculovirus infection have interacted with the physiology of the larval host in unanticipated ways. Sometimes, this interaction is beneficial in that it enhances the pest control properties of the insecticidal protein, and sometimes the interaction is negative in that it reduces the effectiveness of the protein. While it is desirable to exploit or ameliorate the effects of this interaction, doing so has not always been straightforward or possible.

Baculovirus expression of arthropod venom neurotoxins has benefited from advantageous interactions with host physiology. Even when expression levels of neurotoxins are low or undetectable in tissue culture, recombinant viruses that suffer from low neurotoxin expression still sometimes cause paralysis in larvae [40]. This observation extends to expression levels in infected larvae. A recombinant virus expressing LqhIT1 from the early *p35* promoter did not produce levels of LqhIT1 that were detectable by western blot, yet larvae injected with this virus exhibited paralysis [49]. Similarly, while AaIT could be easily detected by western blot in the hemolymph of *H. virescens* larvae infected with an AaIT-expressing recombinant virus, the neurotoxin was barely visible in western blots of hemolymph from infected larvae of the European corn borer, *Ostrinia nubilalis*, even after AaIT in the hemolymph samples was concentrated by immunoprecipitation [53]. Nevertheless, significant reductions in survival time were observed with recombinant virus-infected *O. nubilalis* larvae. Research on the localization and binding of toxic neuropeptides in toxin-injected larvae and larvae infected with toxin-expressing recombinant baculovirus indicated that venom neurotoxins injected in the hemolymph normally bind only at the sites of neuromuscular junctions [122], while baculoviruses are able to infect glial cells and trachea surrounding axons and express and secrete neurotoxins into the channel between the glia and the axons [123]. The neurotoxins are thus able to bind to ion channels all along the length of axons adjacent to infected glial cells. The effectiveness of the toxin is thus enhanced by baculovirus expression, a phenomenon referred to as toxin potentiation.

Toxin expression in baculovirus-infected larvae also results in larvae falling from their position on a plant and dying on the ground, while wild-type baculovirus-infected larvae tend to climb and die in a higher position on a plant [124,125]. The climbing behavior of baculovirus-infected larvae is thought to augment the distribution of progeny OBs from a cadaver and facilitate dissemination [126]. Thus, the final position of recombinant virus-killed larvae may result in a reduction in horizontal transmission, and a higher quantity of recombinant virus OBs present in the soil.

Initial efforts to use JHE as an insecticidal protein were disappointing. Later research disclosed evidence of JHE being taken up and degraded in the pericardial cells of infected hosts [127]. In an effort to boost the half-life of JHE in larval host hemolymph, two lysine residues on JHE predicted to be associated with lysosomal targeting and degradation were replaced with arginine by site-directed mutagenesis [128]. A recombinant virus that encoded a JHE gene with both lysines mutated to arginine (JHE-KK) was found to exhibit a much-improved speed of kill in bioassays and reduced larval feeding relative to wild-type virus-infected larvae [73,128]. The JHE-KK mutant enzyme did not have an increased half-life in hemolymph compared to wild-type JHE, but significantly less JHE-KK was found in the lysosomes of pericardial cells compared to wild-type JHE or JHE forms where only one lysine had been mutated [128].

It is generally assumed that larvae infected with a recombinant baculovirus that expresses an insecticidal protein tend to die from the protein being expressed, or from the combined effects of the insectidal protein and the pathology caused by the viral infection. Host death occurs before the virus is able to complete its life cycle in the host. Thus, it is not surprising that a number of laboratories have observed that larvae infected with fast-killing recombinant baculoviruses tend to produce significantly reduced numbers of progeny OBs [29,81,129]. Because large-scale production of OBs for baculovirus pesticides is carried out using host larvae, reduced OB production by recombinant baculoviruses poses a problem for commercial production of biopesticides using recombinant viruses. One way around this problem is to use a cell culture-based system for large-scale baculovirus OB production. Although there has been much research on the development of such systems [15], no baculovirus pesticide to date has been produced for the market using cell culture. Researchers at DuPont developed a means to suppress expression of genes encoding insecticidal proteins during baculovirus infection by placing the insecticidal gene under the control of tetracycline-repressible regulatory sequences, such that the insecticidal protein was not produced in the presence of a tetracycline analog [130]. Simply adding this analog to the virus inoculum prior to applying to diet surface suppresses expression of the insecticidal protein, leading to a viral infection that results in normal levels of OBs in virus-killed larvae.

Finally, in some studies in which bioassays were conducted against more than one larval instar (developmental stage) or host species, differences in the trends regarding the degree of improvement achieved with expression of an insecticidal peptide or protein were noted among different larval instars and species [46,86]. The reasons for the observed differences in such studies are unclear, and a systematic exploration of how different developmental stages and host species respond to baculovirus expression of any given insecticidal peptide or protein remains to be carried out.

## 4. Looking Forward: Insecticidal RNAs?

While the focus of this review has been on the baculovirus-mediated expression and delivery of insecticidal proteins in pest insects, two studies report the production of recombinant baculoviruses where the insecticidal molecules produced by the viruses consisted of RNA. In these studies, full-length or partial coding sequences for JHE and human *c-myc* were inserted into baculoviruses behind very late promoters in an anti-sense orientation [113,131]. In virus-infected *S. frugiperda* tissue culture cells, a protein species that reacted with c-Myc antibodies disappeared more rapidly from cells infected with the antisense RNA-producing virus than wild-type virus, and expression of the *c-myc* antisense RNA correlated with reduced feeding and survival time in infected larvae [131]. Infection of larvae with a virus expressing antisense JHE RNA resulted in reduced hemolymph JHE titers compared to infection with viruses that lacked any JHE sequences, and approximately 25% of the antisense RNA-producing virus-infected larvae underwent an abortive larva-pupa molt [132].

It is not entirely clear how the antisense RNA expression in the above recombinant viruses mediated reductions in gene expression. The apparent explanation is that gene expression was shut down by RNA interference (RNAi), a method of post-transcriptional gene silencing in which double-stranded RNA triggers the degradation of specific transcripts [133]. Specifically, the *c-myc* and JHE antisense RNAs may have formed duplexes with sense transcripts for Myc-like proteins and JHE, and these RNA duplexes would have been processed into 20–23 bp duplexes. One strand of these short RNA duplexes would then be assembled into an RNA-induced silencing complex (RISC). RNAs complementary to the RISC–associated RNA would then be targeted for destabilization or repression of translation.

Regardless of the actual mechanism by which antisense RNAs eliminated gene expression in cells infected with the antisense-*c-myc* and –JHE recombinant viruses, their example suggests a means for improving baculovirus insecticidal efficacy through the expression of hairpin-loop or double-stranded RNAs that serve as RNAi triggers. This approach could be used to target any gene in the host for silencing. The gene targeted for silencing could be involved in defense response to baculovirus infection. In addition to genes associated with resistance to baculovirus infection that already have been identified [134,135], transcriptomic analyses of baculovirus-infected hosts is expected to reveal the genetic networks involved in response to baculovirus infection [136]. Targeting such genes could conceivably lower the dose of virus required to kill host larvae, or even expand the host range of the virus expressing the RNAi trigger, perhaps by eliminating immune responses that block transmission of baculovirus infection beyond the midgut [137,138,139,140]. Also, the RNAi trigger could itself be insecticidal, by shutting down expression of a gene required for host viability [141]. The attempts at using RNAi to shut down gene expression in lepidopteran larvae have had mixed results [142]. However, in a few cases, feeding RNAi triggers to insects resulted in gene silencing in tissues beyond the gut sheath, indicating that it is possible under some circumstances for the silencing signal to be propagated to other tissues [143,144,145]. By producing an RNAi trigger directly in a cell, baculoviruses circumvent problems with oral delivery of RNAi triggers [146], which may make the establishment of a transmissible RNAi signal more efficient. With the use of an early or cellular promoter to drive expression of the RNAi trigger, a systemic RNAi-mediated suppression of target genes could conceivably occur even if baculovirus infection fails to proceed beyond the midgut.

## 5. Conclusions

A great deal of effort was expended towards the commercial development of biopesticides utilizing genetically modified baculoviruses expressing insecticidal proteins. A large amount of data on efficacy, field performance, and safety of recombinant viruses was generated in the process, by academic, government, and corporate laboratories. This review has summarized the research on the various types of insecticidal proteins that were evaluated for improvement of baculovirus control efficacy, and the factors that control the expression of these proteins.

Unfortunately, the pesticide-manufacturing companies in the USA that were involved in bringing a recombinant baculovirus biopesticide to market ceased their activities and did not complete the registration process for their recombinant viruses. The reasons behind these decisions have not been formally or fully explained, but likely involved the prospects of competing pest control technologies. With increasing restrictions on chemical pesticides and difficulties with achieving public acceptance of genetically modified plants in some parts of the world, naturally occurring insect pathogens such as baculoviruses have become more popular pest control options, and there has been more interest recently in commercial development of pesticides based on these pathogens. This trend hopefully will lead to a renewed interest in recombinant insecticidal protein-expressing baculoviruses and the exploitation of the discoveries summarized in this review.
